# Comparison of Dental Care Visits Before and After Adoption of a Policy to Expand the Dental Workforce in Minnesota

**DOI:** 10.1001/jamahealthforum.2022.0158

**Published:** 2022-03-18

**Authors:** Hawazin W. Elani, Elizabeth Mertz, Ichiro Kawachi

**Affiliations:** Department of Oral Health Policy and Epidemiology, Harvard School of Dental Medicine, Boston, Massachusetts; Department of Health Policy and Management, Harvard T.H. Chan School of Public Health, Boston, Massachusetts; Preventive and Restorative Dental Sciences, Healthforce Center, Philip R. Lee Institute for Health Policy Studies, University of California, San Francisco School of Dentistry, San Francisco; Department of Social and Behavioral Sciences, Harvard T.H. Chan School of Public Health, Boston, Massachusetts

## Abstract

**IMPORTANCE:**

Currently, 13 states and tribal nations have expanded their dental workforce by adopting use of dental therapists. To date, there has been no evaluation of the influence of this policy on dental care use.

**OBJECTIVE:**

To assess changes in dental care use in Minnesota after the implementation of the policy to authorize dental therapists in 2009.

**DESIGN, SETTING, AND PARTICIPANTS:**

In this cross-sectional study of 2 613 716 adults aged 18 years and older, a synthetic control method was used to compare changes in dental care use after the authorization of the policy in Minnesota relative to a synthetic control of nonadopting states. Data from the Behavioral Risk Factor Surveillance System from 2006 to 2018 were analyzed. Data analysis was conducted from June 1, 2021, to December 18, 2021.

**EXPOSURE:**

Authorization of dental therapy.

**MAIN OUTCOMES AND MEASURES:**

Self-reported indicator for whether a respondent had visited a dentist or a dental clinic in the past 12 months.

**RESULTS:**

Among 2 613 716 adults aged 18 years or older, the mean (SD) age at baseline was 46.0 (17.7) years, 396 501 were women (weighted percentage, 51.3%), 503 197 were White (weighted percentage, 67.9%), 54 568 were Black (weighted percentage, 10.1%), 39 282 were Hispanic (weighted percentage, 14.5%), and 34 739 were other race (weighted percentage, 6.7%). The proportion of adults visiting a dentist before the authorization of dental therapists in Minnesota was 76.2% (95% CI, 75.0%-77.4%) in the full sample, 61.5% (95% CI, 58.4%-64.6%) for low-income adults, and 58.4% (95% CI, 53.0%-63.5%) among Medicaid-eligible adults. Authorizing dental therapists in Minnesota was associated with an increase of 7.3 percentage points (95% CI, 5.0-9.5 percentage points) in dental care use among low-income adults, a relative increase of 12.5% (95% CI, 8.6%-16.4%), and an increase of 6.2 percentage points (95% CI, 2.4-10.0 percentage points) among Medicaid-eligible adults, a relative increase of 10.5% (95% CI, 3.9%-17.0%). In addition, the policy was associated with an increase in dental visits among White adults (low-income sample, 10.8 percentage points [95% CI, 8.5-13.0 percentage points]; Medicaid sample, 13.5 percentage points [95% CI, 9.1-17.9 percentage points]), with no corresponding increases among other racial and ethnic groups in the low-income and Medicaid population.

**CONCLUSIONS AND RELEVANCE:**

In this cross-sectional study, expanding the dental workforce through authorization of dental therapists appeared to be associated with an increase in dental visits. In Minnesota, the policy was associated with improved access to dental care among low-income adults overall. However, racial and ethnic disparities in dental use persist.

## Introduction

Despite policy efforts to reduce disparities in oral health, uptake of dental services remains low for low-income and minority populations.^1,2^ A long-standing concern is the capacity of the current dental delivery system to serve the needs of vulnerable populations.^3,4^ In 2020, it was estimated that nearly 60 million US residents lived in areas with dental health professional shortages, indicating a severe maldistribution of the dental workforce.^5^

Increasing the scope of practice of existing health care professionals^6^ or adding new types of clinicians is a core state policy strategy to address clinician shortages and enhance access to health care. In the area of oral health, dozens of states have liberalized the scope of practice^7^ for the long-established occupation of dental hygiene, whereas other states have sought to expand the dental workforce to include dental therapists.^8^ Dental therapists are primary dental care providers who can evaluate and treat basic dental conditions under the supervision of a dentist.^8^ Dental therapists were first introduced in New Zealand in 1921 and have been practicing in more than 50 countries.^9^ In the United States, the Alaska Native Tribal Health Consortium was the first body to authorize dental therapists to practice in 2004; as of 2020, 13 states and tribal nations had authorized dental therapists, and many others are considering it.^8,10-12^

The primary aim of adding dental therapists to the dental team is to improve the affordability of dental care to expand access for underserved populations, particularly low-income and uninsured individuals in rural and tribal areas.^10^ Critics of the policy have raised concerns about the quality of care provided by dental therapists and the overall influence on population oral health.^10,13-15^ Although a number of rigorous studies have demonstrated the clinical competence, patient acceptance, and cost-effectiveness of dental therapists, evidence regarding their influence on dental service use, particularly in the United States, remains sparse.^9,15-17^ This situation is in part due to the small number of individuals practicing (approximately 150 in all) with the majority of dental therapists practicing in Minnesota.^11^

Regardless of the debate over dental therapists, the adoption of this model is increasing, and it is emblematic of states’ willingness to adopt scope-of-practice policies as a key strategy to eliminate disparities in oral health care access. Seven states recently authorized dental therapy; Arizona and Michigan adopted the policy in 2018, and Connecticut, Idaho, Montana, Nevada, and New Mexico adopted it in 2019.^12^ In 2009, Minnesota authorized dental therapists to practice in underserved communities, including health professional shortage areas and in settings with at least 50% of patients with Medicaid coverage or uninsured.^11,12^ The aim of this study was to examine whether authorizing dental therapists was associated with improved dental care use. Our hypothesis was that dental therapists, although few in terms of measuring direct influence, can be used as a proxy for gauging attention to the issue and willingness to innovate at the state level. We took advantage of a natural policy experiment created by state variations in adopting the policy to compare changes in access to dental care in Minnesota with nonauthorization states. We examined changes among adults overall as well as among low-income populations, including Medicaid-eligible adults.

## Methods

### Study Design

This cross-sectional study used a synthetic control approach to construct a counterfactual control population to estimate the association of authorizing dental therapists in Minnesota with dental care use after the authorization of the policy.^18^ The synthetic control method uses a data-driven procedure using data on the outcome and its predictors from before the intervention to create a weighted average of the control units (the synthetic control) that resembles the intervention group in the preintervention period.^18,19^ This process enables comparison of changes in dental visits in Minnesota after the policy to what would have happened there in the absence of the policy.

The synthetic control approach is similar to the difference-in-differences design, which is a common quasi-experimental design used to examine policy effects. However, it requires fewer assumptions and also controls for unmeasured time-varying factors.^20^

### Data and Study Sample

We used data from the Behavioral Risk Factor Surveillance System (BRFSS).^21^ The BRFSS is the largest household annual telephone survey in the world and collects information on participants’ health conditions, health-related risk behaviors, and use of preventive services. We used data from 2006 to 2018 from the BRFSS that includes oral health information. Oral health-related questions are included in the BRFSS every other year. Thus, the study period included BRFSS surveys from 2006 and 2008 (prepolicy years) and 2010, 2012, 2014, 2016, and 2018 (postpolicy years). Participants self-identified their race and ethnicity, which we used to examine racial and ethnic disparities in dental care use. Because we had to create a synthetic control for each comparison, we collapsed non-White racial and ethnic groups into 1 group, non-White.

We excluded from this analysis other states that authorized dental therapists during the study period: Alaska, Arizona, Maine, Michigan, Oregon, Vermont, and Washington. We examined changes in the full sample, low-income population, and Medicaid population. The full sample included all adults aged 18 years or older. The low-income sample included adults aged 18 years or older with family income below 200% of the federal poverty level.^22^ The Medicaid population was limited to the Medicaid-eligible group and thus included adults aged 19 to 64 years with family income up to 138% of the federal poverty level. We estimated the percentage of the federal poverty level according to household size, family income, and the federal poverty guideline for each year.^2^ Our study outcome was measured with a self-reported binary indicator for whether a respondent had visited a dentist or a dental clinic in the past 12 months.

This study used deidentified data and was determined not to be human participant research by the institutional review board of the Harvard Faculty of Medicine; informed patient consent was therefore not obtained. We followed the Strengthening the Reporting of Observational Studies in Epidemiology (STROBE) reporting guideline.

### Statistical Analysis

We created separate synthetic controls for each group examined (full sample, low-income adults, and Medicaid-eligible adults). For the full sample and low-income samples, the donor pool included 42 states, as well as the District of Columbia, that did not authorize dental therapists during the study period (Alabama, Arkansas, California, Colorado, Connecticut, Delaware, Florida, Georgia, Hawaii, Idaho, Illinois, Indiana, Iowa, Kansas, Kentucky, Louisiana, Maryland, Massachusetts, Mississippi, Missouri, Montana, Nebraska, Nevada, New Hampshire, New Jersey, New Mexico, New York, North Carolina, North Dakota, Ohio, Oklahoma, Pennsylvania, Rhode Island, South Carolina, South Dakota, Tennessee, Texas, Utah, Virginia, West Virginia, Wisconsin, and Wyoming).

The synthetic control method relies on the assumption that the treated and donor pool units are similar.^18,19^ Therefore, to construct a suitable synthetic control for the Medicaid sample, we restricted the donor pool to nonadopting states that provide adult dental benefits in Medicaid because Minnesota provides coverage of adult dental benefits through Medicaid. We defined states offering more than emergency dental services to adults through Medicaid as providing dental benefits.^1,23,24^ Accordingly, in the Medicaid analysis, we excluded from the donor pool states that do not cover adult dental services or states that changed their coverage of adult dental benefits through Medicaid during the study period. The donor pool for the Medicaid sample included the following 18 states and the District of Columbia: Arkansas, Connecticut, Indiana, Iowa, Kentucky, Massachusetts, Nebraska, New Jersey, New Mexico, New York, North Carolina, North Dakota, Ohio, Pennsylvania, Rhode Island, South Dakota, Wisconsin, and Wyoming.

In addition, we conducted subgroup analysis to examine racial and ethnic disparities in access to dental care and to assess whether the policy was differentially associated with changes in care among adults in racial and ethnic minority groups. Therefore, we constructed additional synthetic controls for White and non-White individuals in each population examined.

The synthetic control method used an optimization procedure with data on outcome trends and predictor variables in the preintervention period to construct a weighted average of control states from the donor pool to closely match Minnesota before the policy implementation.^18^ We included trends in dental care use and several variables associated with dental care use, including age, age squared, education, race and ethnicity, and the number of dentists per capita in each state.^25^ As a sensitivity analysis, we included other variables associated with dental care use, such as health insurance status, unemployment rate, and rurality, but these variables did not affect or improve the preintervention fit.

To assess the goodness of fit of the synthetic control, we examined prepolicy trends in dental care use in Minnesota and the synthetic control by visually inspecting trends in the preintervention period. We also calculated the root mean square prediction error in the preintervention period, which measured the difference in the path of the outcome between Minnesota and its synthetic control^19^; thus, a small error indicated a good fit between the treatment unit and its synthetic control.

To compare changes between Minnesota and the synthetic control after the policy change, we used Taylor series linearization to estimate differences in having a dental visit and to calculate the 95% CIs, similar to prior literature using synthetic control methods.^26,27^

Finally, as a robustness check for our study design, we performed a placebo test.^18-20^ Here, we repeated the synthetic control analysis but treated each state in the donor pool as the treatment unit. The difference between the actual treated unit and its synthetic control should be larger than that of most donor states in the posttreatment period.^18-20^

We used Stata, version 15.2 (StataCorp LLC), including synth_runner and allsynth packages, for all analyses.^28-31^ We used BRFSS survey weights to account for the survey design. Statistical significance was based on 2-sided *P* ≤ .05, which we calculated with Stata’s postestimation margins options. Data were analyzed from June 1, 2021, to December 18, 2021.

## Results

The full sample included 2 613 716 adults, the low-income sample included 570 487 adults, and the Medicaid sample included 97 383 adults. The mean age of the sample at baseline was 46.0 years (SD, 17.7 years); 396 501 were women (weighted percentage, 51.3%) and 241 250 were men (weighted percentage, 48.7%); and 503 197 were White (weighted percentage, 67.9%), 54 568 were Black (weighted percentage, 10.1%), 39 282 were Hispanic (weighted percentage, 14.5%), and 34 739 were other race (weighted percentage, 6.7%). The prevalence of dental visits in Minnesota before authorization of dental therapists was 76.2% (95% CI, 75.0%-77.4%) in the full sample, 61.5% (95% CI, 58.4%-64.6%) among low-income adults, and 58.4% (95% CI, 53.0%-63.5%) among Medicaid-eligible adults.

### Synthetic Control Goodness of Fit

States contributed differently to each synthetic control. In the full sample (eTable 1 in the Supplement), Rhode Island and Wisconsin contributed almost equally to the synthetic control (analytic weights were 0.521 and 0.479, respectively). Wisconsin had the largest weight in the White subpopulation (0.657), and Rhode Island had the largest weight in the non-White subpopulation (0.695).

In the low-income sample (eTable 2 in the Supplement), Rhode Island had the largest weight for all adults (0.754) and for the White subpopulation (0.593). In the non-White subpopulation, Massachusetts and North Dakota contributed similarly (0.594 and 0.406, respectively) (eTable 2 in the Supplement).

In the Medicaid sample (eTable 3 in the Supplement), Massachusetts had the largest weight for the population of all adults (0.372) and for the non-White subpopulation (0.830). Rhode Island had the largest weight for the White subsample (0.266).

The mean rate values in the prepolicy period for the prevalence of dental care use and its predictors in Minnesota, synthetic Minnesota, and the average of all control states in each donor pool are shown in eTables 4, 5, and 6 in the Supplement. The tables indicate that Minnesota matched the synthetic controls well in terms of the prevalence of dental visits and most predictors in the prepolicy period. For example, in the full sample before the policy adoption, the prevalence of dental visits was much lower in the average control group than in Minnesota. The synthetic control, in contrast, provided values similar to those of actual Minnesota. There was some divergence between Minnesota and the synthetic control in the Hispanic composition for the low-income and Medicaid samples.

**Figure 1.**
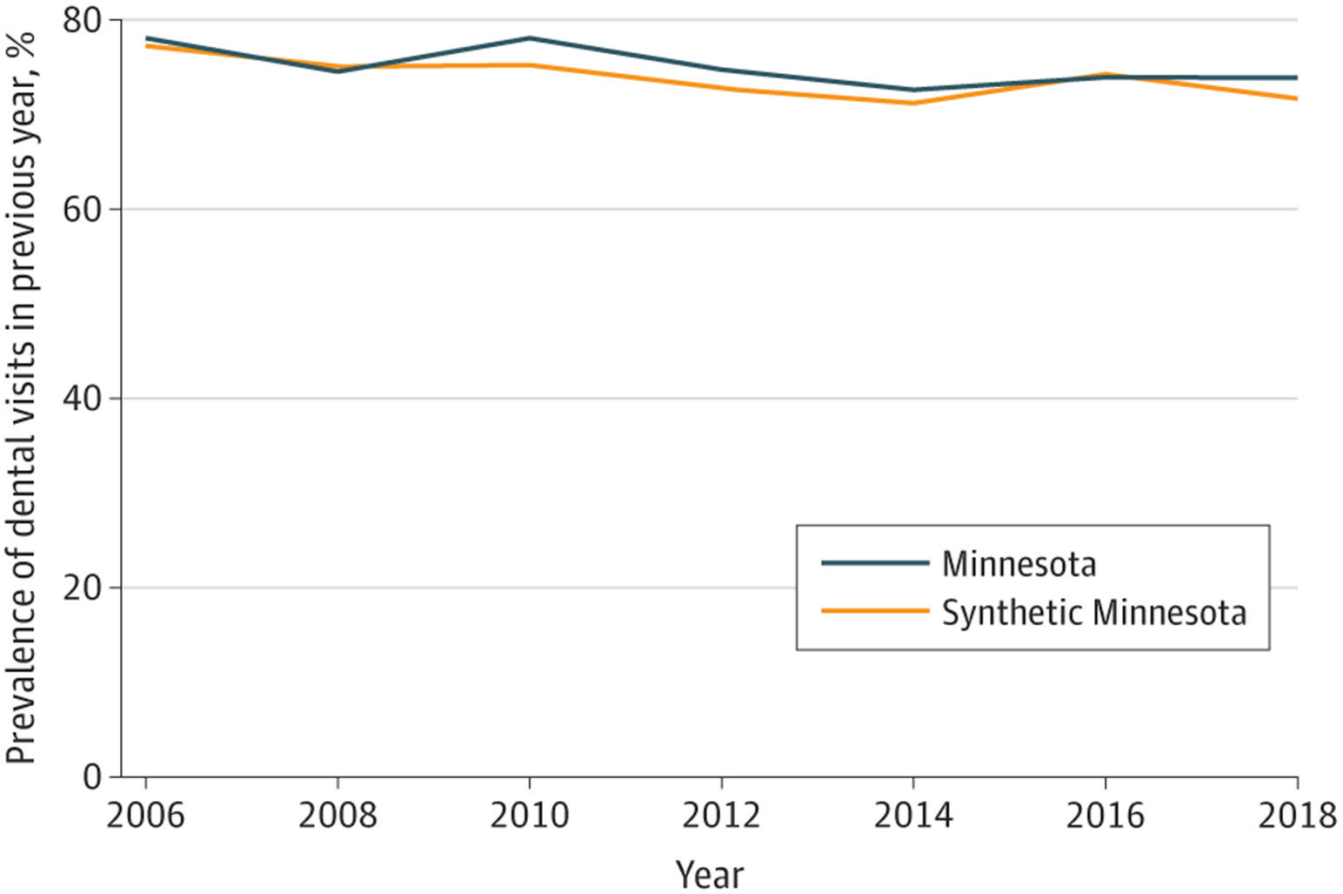
Trends in Access to Dental Care Among All Adults in Minnesota vs Synthetic Control States in the Full Sample Analysis is based on Behavioral Risk Factor Surveillance System data from 2006 to 2018. Dental therapy was adopted in Minnesota in 2009. Refer to eTables 1 through 6 in the Supplement for a description of constructing the synthetic control for each sample.

**Figure 2.**
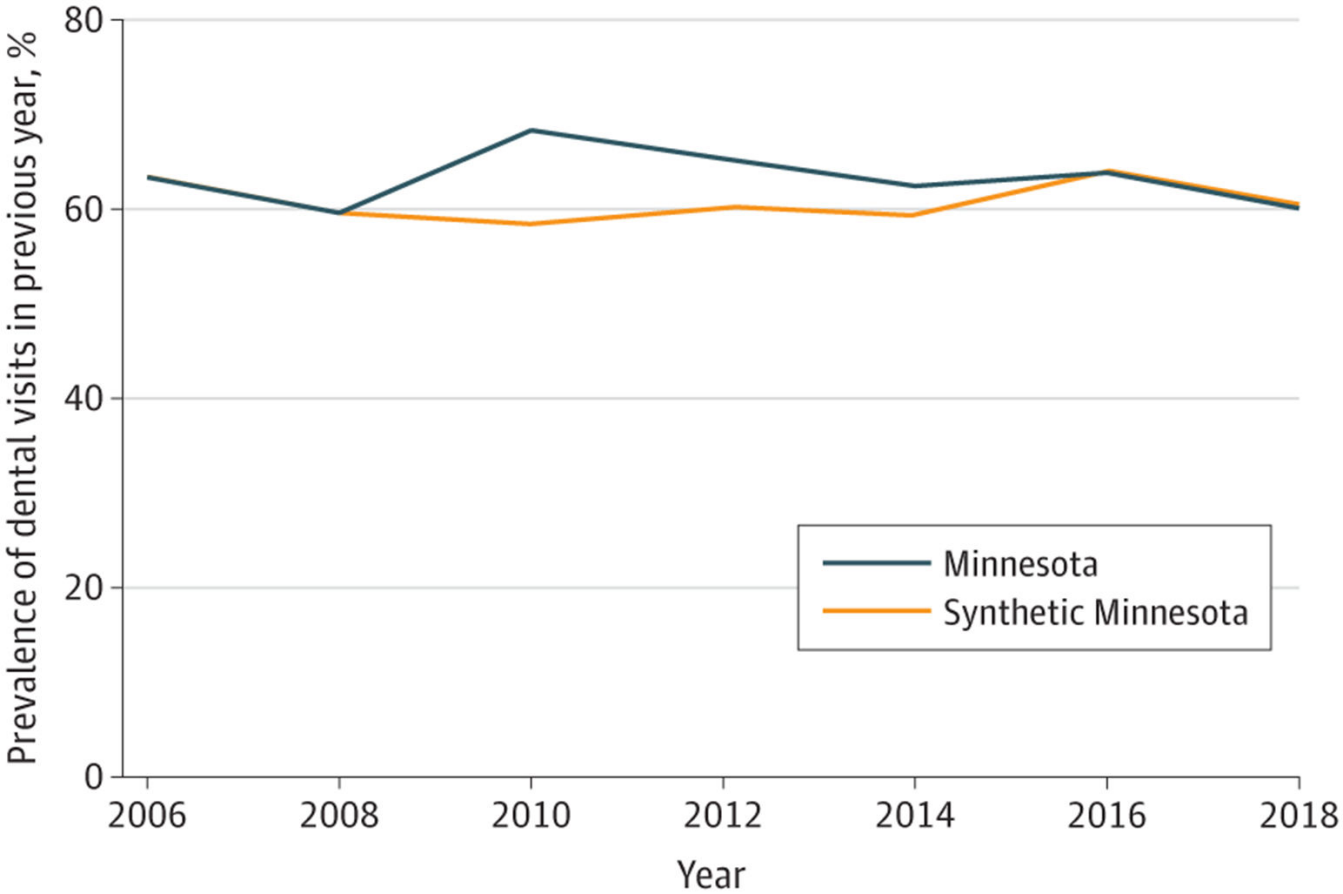
Trends in Access to Dental Care Among All Adults in Minnesota vs Synthetic Control States in the Low-Income Sample Analysis is based on Behavioral Risk Factor Surveillance System data from 2006 to 2018. Dental therapy was adopted in Minnesota in 2009. Refer to eTables 1 through 6 in the Supplement for a description of constructing the synthetic control for each sample.

**Figure 3.**
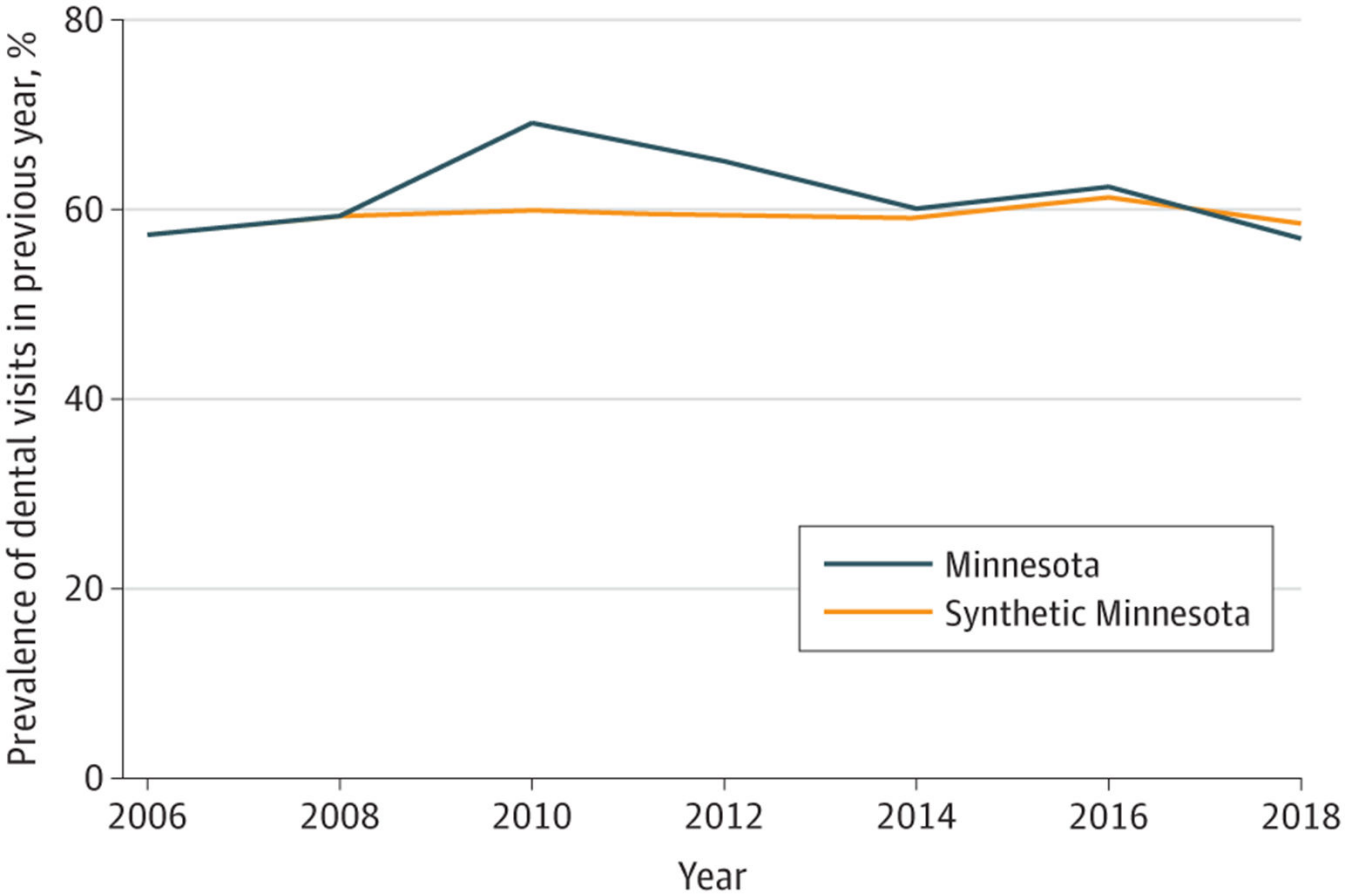
Trends in Access to Dental Care Among All Adults in Minnesota vs Synthetic Control States in the Medicaid Sample Analysis is based on Behavioral Risk Factor Surveillance System data from 2006 to 2018. Dental therapy was adopted in Minnesota in 2009. Refer to eTables 1 through 6 in the Supplement for a description of constructing the synthetic control for each sample.

Trends in the prevalence of reporting a dental visit in the previous year in Minnesota and the synthetic control over time are shown for the full sample (Figure 1), low-income sample (Figure 2), and Medicaid sample (Figure 3). The figures show that, in the prepolicy period (2006-2008), Minnesota closely tracked the synthetic control, indicating an overall good fit and thus providing a suitable counterfactual control population. After 2008, the trends in dental visits diverged, with higher prevalence in dental visits in Minnesota compared with the synthetic control until 2016, when the prevalence in Minnesota decreased below its respective synthetic control, particularly in the low-income and Medicaid groups. The root mean square prediction error for the full, low-income, and Medicaid samples in the preintervention period were all small, suggesting low error and a good preintervention fit between Minnesota and each synthetic control (eTables 1-3 in the Supplement).

### Changes in Access to Dental Care

#### Full Sample

Authorizing dental therapists in Minnesota was associated with an increase of 2.3 percentage points (95% CI, 1.5-3.1 percentage points) in the prevalence of having a dental visit in Minnesota relative to its synthetic control, a relative increase of 3.2% (95% CI, 2.0%-4.4%) (Table). In the subgroup analysis, we estimated an increase of 2.0 percentage points (95% CI, 1.0-2.9 percentage points) in dental visits among White adults and an increase of 5.0 percentage points (95% CI, 3.5-6.5 percentage points) among non-White adults associated with the policy for 2.6% (95% CI, 1.4%-3.9%) and 8.4% (95% CI, 5.8%-11.0%) relative increases, respectively. However, in the placebo analysis, the magnitude of the difference for several placebo states (16 of 43 tests) was larger than what we estimated for Minnesota compared with its synthetic control (eFigure 1 in the Supplement). Therefore, our estimates from the synthetic control did not provide significant evidence for changes in dental visits for the full sample after policy implementation.

#### Low-Income Adults

The absolute difference in the prevalence of dental visits between Minnesota and its synthetic control after policy implementation was 7.3 percentage points (95% CI, 5.0-9.5 percentage points), a relative increase of 12.5% (95% CI, 8.6%-16.4%). In the subgroup analysis, we estimated that authorizing dental therapists was associated with an increase of 10.8 percentage points (95% CI, 8.5-13.0 percentage points) in dental visits for White adults (relative increase, 19.8%; 95% CI, 15.6%-24.1%). However, we did not detect any significant changes for non-White adults. The synthetic control estimates were robust in placebo analysis, indicating significant improvements in dental visits after the adoption of dental therapy in Minnesota until 2014. No other state in the donor pool demonstrated a greater gap than what we observed in Minnesota in 2010 (0 of 43 states), and only a small number of states had a larger gap in 2012 (2 of 43 states) and 2014 (6 of 43 states) (eFigure 2 in the Supplement).

#### Medicaid-Eligible Adults

Authorizing dental therapists in Minnesota was associated with an increase of 6.2 percentage points (95% CI, 2.4-10.0 percentage points) in having a dental visit for all adults and an increase of 13.5 percentage points (95% CI, 9.1-17.9 percentage points) for White adults (10.5% [95% CI, 3.9%-17.0%] and 25.5% [95% CI, 17.0%-34.1%] relative increase, respectively). Results from the placebo test are consistent with our observed synthetic control estimates, suggesting that our findings are unlikely to be due to chance. The magnitude of the gap between Minnesota and its synthetic control was larger than that of all other states in the donor pool until 2012 (0 of 19 states), and only a few states had a larger gap in 2014 (6 of 19 states) (eFigure 3 in the Supplement).

## Discussion

Despite previous research demonstrating the effectiveness of dental therapists in the United States,^9,16,17^ little is yet known about the policy influence on populations’ access to dental care. Much of the evidence is based on small observational studies limited to a single state.^32,33^ Using a synthetic control method and nationally representative data, we examined the association between authorizing dental therapists and dental visits by comparing Minnesota with a synthetic control. We found that authorizing dental therapy was associated with increases in dental visits among low-income and Medicaid-eligible adults overall. We also found that the adoption of the policy was associated with an increase in dental care use for White adults without corresponding increases among other racial and ethnic groups. These findings provide new evidence on the association between authorizing dental therapists and access to dental care.

Among the 13 states that have authorized dental therapists, 8 are still in the implementation stage and do not have any dental therapists in practice. Recent estimates suggest there are approximately 150 dental therapists practicing in the United States, with nearly 100 therapists practicing in Minnesota.^10,11^ The increases in dental use observed in our study may be owing to a combination of a direct association with the newly deployed workforce and a positive spillover effect of the policy on dentists. In addition to a dentist shortage in underserved regions, low dentist participation in Medicaid creates a major barrier to accessing dental care among Medicaid beneficiaries.^4,34^ We speculate that authorization of dental therapists created competition in the dental industry, encouraging more dentists to be willing to treat low-income and underserved populations.^35^

Our findings suggest that barriers to obtaining dental care remain a significant challenge for minority populations and underscore the importance of predisposing factors on the use of health care services.^36^ Estimates from our analysis indicated significant improvements in having a dental visit in the past year associated with the policy but only for White adults. These findings have important implications for state policies and are likely associated with structural racism in health care, such as differential distribution and segregation of dental clinics.^37^

Our study extends previous findings regarding the influence of expanding the scope of practice or adding new health care professionals on health care delivery. Several studies have shown that nurse practitioners play an important role in improving access and health outcomes for underserved populations, particularly in rural areas.^38,39^ In addition, there is evidence indicating greater acceptance of Medicaid beneficiaries in health care settings that include nurse practitioners.^40^ Other studies have also suggested that expanding dental hygienists’ scope of practice facilitates the delivery of preventive oral health services, leading to better population oral health.^6,41^

Successful integration of dental therapists into dental care delivery systems requires a collaborative effort between policy makers, clinicians, and dental educators. As the availability of training programs and the number of dental therapists increase, future research should continue to track and monitor the influence of this evolving workforce model on population oral health.

### Limitations

This study has limitations. We measured access to dental care by using only 1 self-reported question, which is susceptible to both recall and social desirability biases. In addition, the survey question asked whether participants visited a dental clinic and not whether they had consulted a dental therapist. Therefore, in Minnesota, some respondents who were treated by a dental therapist might have answered no to the question about visiting a dentist. Hence, we might have slightly underestimated the actual number of people who received dental care (albeit from a dental therapist).

In addition, we did not assess the association between adopting dental therapy and oral health outcomes. As more data become available, future research should assess changes in use and clinical measures of oral health associated with the policy.

Finally, estimates from the synthetic comparison involved few states; for example, in the full sample, only 2 states contributed to the synthetic control. This limitation may have reduced the presumed benefit of synthesis for generating a control estimate that averages the noise associated with interstate variation.

## Conclusions

Evidence from this study suggests that expanding the dental workforce to include dental therapists in Minnesota shows associated improvements in access to dental care among disadvantaged populations. Our study also strengthens the evidence on persistent racial and ethnic disparities in access to dental care. As more dental therapists begin practice, it remains important to conduct more research to examine mechanisms by which workforce policies can improve access to dental care to meet the oral health needs of underserved communities.

## Supplementary Material

Supplemental Online Content **eTable 1.** State Weights in Synthetic Minnesota in the Full Sample
**eTable 2.** State Weights in Synthetic Minnesota in the Low-Income Sample
**eTable 3.** State Weights in Synthetic Minnesota in the Medicaid Sample
**eTable 4.** Predictor Balance in the Prepolicy Period in the Full Sample (2006-2008)
**eTable 5.** Predictor Balance in the Prepolicy Period in the Low-Income Sample (2006-2008)
**eTable 6.** Predictor Balance in the Prepolicy Period in the Medicaid Sample (2006-2008)
**eFigure 1.** Placebo Analysis for the Full Sample for All Control States Before and After the Adoption of Dental Therapy in Minnesota in 2009
**eFigure 2.** Placebo Analysis for the Low-Income Sample for All Control States Before and After the Adoption of Dental Therapy in Minnesota in 2009
**eFigure 3.** Placebo Analysis for the Medicaid Sample for All Control States Before and After the Adoption of Dental Therapy in Minnesota in 2009

## Figures and Tables

**Table.** Changes in Dental Visits in Minnesota Associated With Adopting the Use of Dental Therapists Relative to Synthetic Minnesota^a^

	Unadjusted proportion after policy adoption, weighted % (95% CI)		Relative change after policy adoption^b^	
	Minnesota	Synthetic Minnesota	Weighted % (95% CI)	Linear *P* value
Full sample				
All adults	74.6 (74.1 to 75.1)	72.3 (71.6 to 73.0)	3.2 (2.0 to 4.4)	<.001
Race and ethnicity				
Non-White^c^	64.3 (62.8 to 65.9)	59.3 (59.0 to 59.6)	8.4 (5.8 to 11.0)	<.001
White	76.5 (76.0 to 77.0)	74.6 (73.8 to 75.3)	2.6 (1.4 to 3.9)	<.001
Low-income sample				
All adults	65.2 (63.2 to 67.1)	57.9 (56.9 to 58.9)	12.5 (8.6 to 16.4)	<.001
Race and ethnicity				
Non-White^c^	66.0 (60.6 to 71.3)	65.5 (62.8 to 68.3)	0.7 (−8.5 to 9.8)	.89
White	65.0 (62.9 to 67.1)	54.2 (53.4 to 55.1)	19.8 (15.6 to 24.1)	<.001
Medicaid sample				
All adults	65.4 (61.7 to 69.0)	59.1 (57.9 to 60.4)	10.5 (3.9 to 17.0)	.002
Race and ethnicity				
Non-White^c^	62.8 (56.1 to 69.6)	66.3 (62.6 to 70.1)	−5.16 (−3.8 to 6.2)	.37
White	66.3 (62.0 to 70.6)	52.8 (51.7 to 53.9)	25.5 (17.0 to 34.1)	<.001

a Full sample includes adults aged 18 years and older. The low-income sample includes adults aged 18 years and older with a family income below 200% of the federal poverty level. The Medicaid sample includes adults aged 19 to 64 years with a family income up to 138% of the federal poverty level. Refer to eTables 1 through 6 in the Supplement for a description of constructing the synthetic control for each sample.

b Taylor series linearization was used to calculate the 95% CIs.

c Non-White included Black, Hispanic, and other race subgroups.

## Key Points

### Question

Is expansion of the dental workforce by authorizing dental therapists in Minnesota associated with increased dental care use?

### Findings

In this cross-sectional study of 2 613 716 adults, authorizing dental therapists was associated with an increase in dental care use among low-income adults. In subgroup analysis, authorizing dental therapists was associated with an increase in dental visits for White adults, but no significant change in use was detected for other racial and ethnic groups.

### Meaning

Expanding the dental workforce in Minnesota by authorizing dental therapists was associated with improved dental care use, particularly among low-income adults; however, racial and ethnic disparities in dental use persist.

## References

[1] Elani HW Sommers BD Kawachi I. Changes in coverage and access to dental care five years after ACA Medicaid expansion Health Aff (Millwood) 2020;39(11):1900–1908. doi:10.1377/hlthaff.2020.00386 33136492 PMC8056359

[2] Elani HW, Kawachi I, Sommers BD. Medicaid healthy behavior incentives and use of dental services. Health Serv Res 2021;56(4):702–708. doi:10.1111/1475-6773.13672 34008193 PMC8313965

[3] Mertz EA. The dental-medical divide. Health Aff (Millwood). 2016;35(12):2168–2175. doi:10.1377/hlthaff.2016.0886 27920303

[4] Vujicic M. Is the number of Medicaid providers really that important? J Am Dent Assoc. 2016;147(3):221–223. doi:10.1016/j.adaj.2016.01.004 26825375

[5] Kaiser Family Foundation. Dental care health professional shortage areas (HPSAs). Accessed May 26, 2021. https://www.kff.org/other/state-indicator/dental-care-health-professional-shortage-areas-hpsas/?currentTimeframe=0&sortModel=%7B%22colId%22:%22Location%22,%22sort%22:%22asc%22%7D

[6] Langelier M, Continelli T, Moore J, Baker B, Surdu S. Expanded scopes of practice for dental hygienists associated with improved oral health outcomes for adults. Health Aff (Millwood). 2016;35(12):2207–2215. doi:10.1377/hlthaff.2016.0807 27920308

[7] Oral Health Workforce Research Center. Variation in dental hygiene scope of practice by state. Updated January 2019. Accessed August 23, 2021. https://oralhealthworkforce.org/resources/variation-in-dental-hygiene-scope-of-practice-by-state/

[8] Oral Health Workforce Research Center. Authorization status of dental therapists by state. Updated September 2020. Accessed August 23, 2021. https://oralhealthworkforce.org/authorization-status-of-dental-therapists-by-state/

[9] Nash DA, Friedman JW, Mathu-Muju KR, A review of the global literature on dental therapists. Community Dent Oral Epidemiol. 2014;42(1):1–10. doi:10.1111/cdoe.12052 23646862

[10] Mertz E, Self K, Moore J, Maxey H. The oral health workforce. In: Mascarenhas AK, Okunseri C, Dye BA, eds. Burt and Eklund’s Dentistry, Dental Practice, and the Community. WB Saunders; 2021:80–91. doi:10.1016/B978-0-323-55484-8.00008-3

[11] Simon L, Donoff RB, Friedland B. Dental therapy in the United States: are developments at the state level a reason for optimism or a cause for concern? J Public Health Dent. 2021;81(1):12–20. doi:10.1111/jphd.12388 32805762

[12] Mertz E, Kottek A, Werts M, Langelier M, Surdu S, Moore J. Dental therapists in the United States: health equity, advancing. Med Care. 2021;59(suppl 5):S441–S448. doi:10.1097/MLR.0000000000001608 34524241 PMC8428854

[13] Mathu-Muju KR. Chronicling the dental therapist movement in the United States. J Public Health Dent. 2011;71(4):278–288. doi:10.1111/j.1752-7325.2011.00270.x 22320286

[14] Catalanotto F. In defense of dental therapy: an evidence-based workforce approach to improving access to care. J Dent Educ. 2019;83 (2)(suppl):S7–S15. doi:10.21815/JDE.019.036 30709933

[15] Wright JT, Graham F, Hayes C, A systematic review of oral health outcomes produced by dental teams incorporating midlevel providers. J Am Dent Assoc. 2013;144(1):75–91. doi:10.14219/jada.archive.2013.0017 23283929

[16] Mathu-Muju KR, Friedman JW, Nash DA. Current status of adding dental therapists to the oral health workforce in the United States. Current Oral Health Reports. 2016;3(3):147–154. doi:10.1007/s40496-016-0091-1

[17] Chi DL, Lenaker D, Mancl L, Dunbar M, Babb M. Dental therapists linked to improved dental outcomes for Alaska Native communities in the Yukon-Kuskokwim Delta. J Public Health Dent. 2018;78(2):175–182. doi:10.1111/jphd.12263 29377127 PMC6019600

[18] Abadie A, Diamond A, Hainmueller J. Synthetic control methods for comparative case studies: estimating the effect of California’s tobacco control program. J Am Stat Assoc. 2010;105(490):493–505. doi:10.1198/jasa.2009.ap08746

[19] Abadie A, Diamond A, Hainmueller J. Comparative politics and the synthetic control method. Am J Pol Sci. 2015;59(2):495–510. doi:10.1111/ajps.12116

[20] Abadie A, Gardeazabal J. The economic costs of conflict: a case study of the Basque Country. Am Econ Rev. 2003;93(1):113–132. doi:10.1257/000282803321455188

[21] Centers for Disease Control and Prevention. Behavioral Risk Factor Surveillance System. Accessed April 4, 2021. https://www.cdc.gov/brfss/annual_data/annual_data.htm

[22] Kaiser Family Foundation. Expanding health coverage for low-income adults: filling the gaps in Medicaid eligibility. April 30, 2009. Accessed June 5, 2021. https://www.kff.org/health-reform/issue-brief/expanding-health-coverage-for-low-income-adults/

[23] Hinton E, Paradise J. Access to dental care in Medicaid: spotlight on nonelderly adults. Kaiser Family Foundation. March 17, 2016. Accessed April 27, 2021. https://kff.org/medicaid/issue-brief/access-to-dental-care-in-medicaid-spotlight-on-nonelderly-adults/

[24] Medicaid.gov. Medicaid state plan amendments. Accessed March 13, 2021. https://www.medicaid.gov/state-resource-center/medicaid-state-plan-amendments/index.html

[25] American Dental Association, Health Policy Institute. Supply of dentists in the US: 2001-2021. Accessed May 20, 2021. https://www.ada.org/resources/research/health-policy-institute/dentist-workforce

[26] Auty SG, Shafer PR, Dusetzina SB, Griffith KN. Association of Medicaid managed care drug carve outs with hepatitis C virus prescription use. JAMA Health Forum. 2021;2(8):e212285. doi:10.1001/jamahealthforum.2021.2285 35977199 PMC8796891

[27] Auty SG, Shafer PR, Griffith KN. Medicaid subscription-based payment models and implications for access to hepatitis C medications. JAMA Health Forum. 2021;2(8):e212291. doi:10.1001/jamahealthforum.2021.2291 35977192 PMC8796990

[28] Stata Statistical Software: Release 15.1. Version 15.1. StataCorp LLC; 2017.

[29] Abadie A, Diamond A, Hainmueller J. SYNTH: Stata module to implement synthetic control methods for comparative case studies. IDEAS. Accessed December 1, 2021. https://ideas.repec.org/c/boc/bocode/s457334.html

[30] Galiani S, Quistorff B. The synth_runner package: utilities to automate synthetic control estimation using synth. Stata J. 2017;17(4):834–849. doi:10.1177/1536867X1801700404

[31] Wiltshire JC. allsynth: Synthetic control bias-correction utilities for Stata. August 5, 2021. Accessed December 20, 2021. https://www.stata.com/meeting/us21/slides/US21_Wiltshire.pdf

[32] Chi DL, Mancl L, Hopkins S, Supply of care by dental therapists and emergency dental consultations in Alaska Native communities in the Yukon-Kuskokwim Delta: a mixed methods evaluation. Community Dent Health. 2020;37(3):190–198. doi:10.1922/CDH_00022Chi09 32673470

[33] Blue CM, Kaylor MB. Dental therapy practice patterns in Minnesota: a baseline study. Community Dent Oral Epidemiol. 2016;44(5):458–466. doi:10.1111/cdoe.12235 27112771

[34] American Dental Association, Health Policy Institute. Dentist participation in Medicaid or CHIP. Accessed January 27, 2021. https://www.ada.org/-/media/project/ada-organization/ada/ada-org/files/resources/research/hpi/hpigraphic_0820_1.pdf

[35] Yang YT, Chen B, Wanchek T. Dental therapists: a solution to a shortage of dentists in underserved communities? Public Health Rep. 2017;132(3):285–288. doi:10.1177/0033354917698114 28448769 PMC5415248

[36] Andersen RM. Revisiting the behavioral model and access to medical care: does it matter? J Health Soc Behav. 1995;36(1):1–10. doi:10.2307/2137284 7738325

[37] Bailey ZD, Krieger N, Agénor M, Graves J, Linos N, Bassett MT. Structural racism and health inequities in the USA: evidence and interventions. Lancet. 2017;389(10077):1453–1463. doi:10.1016/S0140-6736(17)30569-X 28402827

[38] Barnes H, Richards MR, McHugh MD, Martsolf G. Rural and nonrural primary care physician practices increasingly rely on nurse practitioners. Health Aff (Millwood). 2018;37(6):908–914. doi:10.1377/hlthaff.2017.1158 29863933 PMC6080248

[39] Yang BK, Johantgen ME, Trinkoff AM, Idzik SR, Wince J, Tomlinson C. State nurse practitioner practice regulations and US health care delivery outcomes: a systematic review. Med Care Res Rev. 2021;78(3):183–196. doi:10.1177/1077558719901216 31997710

[40] Barnes H, Richards MR, Martsolf GR, Nikpay SS, McHugh MD. Association between physician practice Medicaid acceptance and employing nurse practitioners and physician assistants: a longitudinal analysis. Health Care Manage Rev. 2022;47(1):21–27. doi:10.1097/HMR.0000000000000291 33181552 PMC8110602

[41] Simmer-Beck M, Walker M, Gadbury-Amyot C, Liu Y, Kelly P, Branson B. Effectiveness of an alternative dental workforce model on the oral health of low-income children in a school-based setting. Am J Public Health. 2015;105(9):1763–1769. doi:10.2105/AJPH.2015.302714 26180957 PMC4539834

