# Supplementary material for: Comparison of Dental Care Visits Before and After Adoption of a Policy to Expand the Dental Workforce in Minnesota
Source: JAMA Health Forum. Author manuscript; Available in PMC 2023 Mar 18. (PMC8933736; doi:10.1001/jamahealthforum.2022.0158)
Supplement: Supplemental Online Content — eTable 1. State Weights in Synthetic Minnesota in the Full Sample eTable 2. State Weights in Synthetic Minnesota in the Low-Income Sample eTable 3. State Weights in Synthetic Minnesota in the Medicaid Sample eTable 4. Predictor Balance in the Prepolicy Period in the Full Sample (2006-2008) eTable 5. Predictor Balance in the Prepolicy Period in the Low-Income Sample (2006-2008) eTable 6. Predictor Balance in the Prepolicy Period in the Medicaid Sample (2006-2008) eFigure 1. Placebo Analysis for the Full Sample for All Control States Before and After the Adoption of Dental Therapy in Minnesota in 2009 eFigure 2. Placebo Analysis for the Low-Income Sample for All Control States Before and After the Adoption of Dental Therapy in Minnesota in 2009 eFigure 3. Placebo Analysis for the Medicaid Sample for All Control States Before and After the Adoption of Dental Therapy in Minnesota in 2009 [file NIHMS1799388-supplement-Supplemental_Online_Content.pdf]

Supplemental Online Content

Elani HW, Mertz E, Kawachi I. Comparison of dental care visits before and after adoption of a policy to expand the dental workforce in Minnesota. *JAMA Health Forum*. 2022;3(3):e220158. doi:10.1001/jamahealthforum.2022.0158

eTable 1. State Weights in Synthetic Minnesota in the Full Sample

eTable 2. State Weights in Synthetic Minnesota in the Low-Income Sample

eTable 3. State Weights in Synthetic Minnesota in the Medicaid Sample

eTable 4. Predictor Balance in the Prepolicy Period in the Full Sample (2006-2008)

eTable 5. Predictor Balance in the Prepolicy Period in the Low-Income Sample (2006-2008)

eTable 6. Predictor Balance in the Prepolicy Period in the Medicaid Sample (2006-2008)

eFigure 1. Placebo Analysis for the Full Sample for All Control States Before and After the Adoption of Dental Therapy in Minnesota in 2009

eFigure 2. Placebo Analysis for the Low-Income Sample for All Control States Before and After the Adoption of Dental Therapy in Minnesota in 2009

eFigure 3. Placebo Analysis for the Medicaid Sample for All Control States Before and After the Adoption of Dental Therapy in Minnesota in 2009

This supplemental material has been provided by the authors to give readers additional information about their work.

© 2022 Elani et al. *JAMA Health Forum.*

eTable 1. State Weights in Synthetic Minnesota in the Full Sample

	Weight		
	All adults	White subpopulation	Non-White subpopulation
**Control state**			
Alabama	0	0	0.013
Arkansas	0	0	0.001
California	0	0	0.003
Colorado	0	0	0.002
Connecticut	0	0	0.002
Delaware	0	0	0.004
District of Columbia	0	0.343	0.077
Florida	0	0	0.002
Georgia	0	0	0.003
Hawaii	0	0	0.099
Idaho	0	0	0.002
Illinois	0	0	0.002
Indiana	0	0	0.001
Iowa	0	0	0.004
Kansas	0	0	0.003
Kentucky	0	0	0.002
Louisiana	0	0	0.002
Maryland	0	0	0.005
Massachusetts	0	0	0
Mississippi	0	0	0.003
Missouri	0	0	0.001
Montana	0	0	0.006
Nebraska	0	0	0.003
Nevada	0	0	0.004
New Hampshire	0	0	0.004
New Jersey	0	0	0.003
New Mexico	0	0	0.003
New York	0	0	0.002
North Carolina	0	0	0.002
North Dakota	0	0	0.005
Ohio	0	0	0.003
Oklahoma	0	0	0.002
Pennsylvania	0	0	0.002
Rhode Island	0.521	0	0.695
South Carolina	0	0	0.002
South Dakota	0	0	0.002
Tennessee	0	0	0.002
Texas	0	0	0.002

This supplemental material has been provided by the authors to give readers additional information about their work.

© 2022 Elani et al. *JAMA Health Forum.*

Utah	0	0	0.002
Virginia	0	0	0.004
West Virginia	0	0	0.007
Wisconsin	0.479	0.657	0.007
Wyoming	0	0	0.006
**Sum**	1	1	1
**Root Mean Square Prediction Error (RMSPE)**	0.69	0.76	3.23e-12

Note. The donor pool for the full sample included 43 states.

eTable 2. State Weights in Synthetic Minnesota in the Low-Income Sample

	Weight		
	All adults	White subpopulation	Non-White subpopulation
**Control state**			
Alabama	0.002	0.062	0
Arkansas	0.001	0.001	0
California	0.001	0.003	0
Colorado	0.001	0.003	0
Connecticut	0.017	0.001	0
Delaware	0.003	0.003	0
District of Columbia	0.108	0.114	0
Florida	0.002	0.004	0
Georgia	0.003	0.008	0
Hawaii	0.005	0.053	0
Idaho	0.001	0.003	0
Illinois	0.001	0.002	0
Indiana	0.001	0.003	0
Iowa	0.002	0.005	0
Kansas	0.001	0.004	0
Kentucky	0.001	0.015	0
Louisiana	0.002	0.001	0
Maryland	0.003	0.004	0
Massachusetts	0.005	0.001	0.594
Mississippi	0.001	0.002	0
Missouri	0.001	0.001	0
Montana	0.001	0.005	0
Nebraska	0.002	0.004	0
Nevada	0.001	0.002	0
New Hampshire	0.002	0.004	0
New Jersey	0.004	0	0
New Mexico	0.001	0.002	0
New York	0.004	0.002	0

Note. The donor pool for the low-income sample included 43 states.

This supplemental material has been provided by the authors to give readers additional information about their work.

© 2022 Elani et al. *JAMA Health Forum.*

North Carolina	0.001	0.004	0
North Dakota	0.002	0.001	0.406
Ohio	0.003	0.009	0
Oklahoma	0	0.002	0
Pennsylvania	0.012	0.004	0
Rhode Island	0.754	0.593	0
South Carolina	0.001	0.004	0
South Dakota	0.002	0.006	0
Tennessee	0.001	0.01	0
Texas	0.001	0.002	0
Utah	0.001	0.004	0
Virginia	0.002	0.003	0
West Virginia	0.001	0.01	0
Wisconsin	0.042	0.032	0
Wyoming	0.001	0.003	0
**Sum**	1	1	1
**Root Mean Square Prediction Error (RMSPE)**	1.52e-11	1.48e-11	1.40

Note. The donor pool for the low-income sample included 43 states.

This supplemental material has been provided by the authors to give readers additional information about their work.

© 2022 Elani et al. *JAMA Health Forum.*

eTable 3. State Weights in Synthetic Minnesota in the Medicaid Sample

	Weight		
	All adults	White subpopulation	Non-White subpopulation
**Control state**			
Arkansas	0.006	0.013	0
Connecticut	0.024	0.051	0
District of Columbia	0.033	0.119	0
Indiana	0.006	0.026	0
Iowa	0.017	0.038	0
Kentucky	0.005	0.017	0
Massachusetts	0.372	0.044	0.83
Nebraska	0.022	0.04	0
New Jersey	0.021	0.041	0
New Mexico	0.01	0.025	0
New York	0.043	0.037	0
North Carolina	0.008	0.022	0
North Dakota	0.035	0.044	0
Ohio	0.019	0.047	0
Pennsylvania	0.017	0.032	0
Rhode Island	0.304	0.266	0
South Dakota	0.023	0.053	0.17
Wisconsin	0.026	0.06	0
Wyoming	0.009	0.025	0
**Sum**	1	1	1
**Root Mean Square Prediction Error (RMSPE)**	1.27e-10	7.94e-12	3.70

Note. The donor pool for Medicaid sample included 19 states.

This supplemental material has been provided by the authors to give readers additional information about their work.

© 2022 Elani et al. *JAMA Health Forum.*

eTable 4. Predictor Balance in the Prepolicy Period in the Full Sample (2006-2008)

	Minnesota	Synthetic Minnesota	Average of control units[a]
**Variable**			
**All adults**			
Age	45.9	46.5	46.2
Age squared	2419.9	2491.3	2447.9
% Black (log)	-3.5	-3.4	-3.1
% Hispanic (log)	-3.9	-3.0	-2.9
% White (log)	-0.09	-0.14	-0.33
% with less than high school degree	1.4	2.2	3.2
Number of dentists per capita	60.1	52.9	56.0
Prevalence of dental visit in previous year, 2006	78.1	77.3	67.9
Prevalence of dental visit in previous year, 2008	74.5	75.1	68.4
**White subpopulation**			
Age	46.8	45.9	47.7
Age squared	2505.16	2414.0	2591.8
% with less than high school degree	1.2	1.03	1.6
Number of dentists per capita	60.1	70.6	56.0
Prevalence of dental visit in previous year, 2006	79.0	78.1	70.9
Prevalence of dental visit in previous year, 2008	75.4	76.0	71.5
**Non-White subpopulation**			
Age	36.7	40.8	41.4
Age squared	1578.9	1956.7	2001.1
% with less than high school degree	2.5	8.3	7.1
Number of dentists per capita	60.1	60.0	56.0
Prevalence of dental visit in previous year, 2006	69.8	69.7	60.0
Prevalence of dental visit in previous year, 2008	64.8	64.7	60.6

Note. Age, age squared, % Black, % Hispanic, % White, % with less than high school degree, and the number of dentists per capita were all averaged for the pre-policy period (2006-2008). [a] Control states for the full sample included 43 states: Alabama, Arkansas, California, Colorado, Connecticut, Delaware, District of Columbia, Florida, Georgia, Hawaii, Idaho, Illinois, Indiana, Iowa, Kansas, Kentucky, Louisiana, Maryland, Massachusetts, Mississippi, Missouri, Montana, Nebraska, Nevada, New Hampshire, New Jersey, New Mexico, New York, North Carolina, North Dakota, Ohio, Oklahoma, Pennsylvania, Rhode Island, South Carolina, South Dakota, Tennessee, Texas, Utah, Virginia, West Virginia, Wisconsin, Wyoming.

This supplemental material has been provided by the authors to give readers additional information about their work.

© 2022 Elani et al. *JAMA Health Forum.*

eTable 5. Predictor Balance in the Prepolicy Period in the Low-Income Sample (2006-2008)

	Minnesota	Synthetic Minnesota	Average of control units[a]
**Variable**			
**All adults**			
Age	44.2	44.5	43.7
Age squared	2367.2	2369.6	2260.5
% Black (log)	-2.5	-2.5	-2.6
% Hispanic (log)	-3.4	-1.8	-2.4
% White (log)	-0.20	-0.64	-0.56
% with less than high school degree	4.1	7.6	6.7
Number of dentists per capita	60.1	60.1	56.0
Prevalence of dental visit in previous year, 2006	63.4	63.4	53.0
Prevalence of dental visit in previous year, 2008	59.6	59.7	52.2
**White subpopulation**			
Age	46.3	46.3	45.9
Age squared	2571.6	2569.8	2499.5
% with less than high school degree	3.8	3.8	3.6
Number of dentists per capita	60.1	60.0	56.0
Prevalence of dental visit in previous year, 2006	63.7	63.6	54.3
Prevalence of dental visit in previous year, 2008	58.5	58.4	53.0
**Non-White subpopulation**			
Age	35.2	37.1	39.9
Age squared	1471.6	1647.3	1872.5
% with less than high school degree	5.1	11.0	10.0
Number of dentists per capita	60.1	64.9	56.0
Prevalence of dental visit in previous year, 2006	62.1	60.9	51.3
Prevalence of dental visit in previous year, 2008	65.3	63.8	51.2

Note. Age, age squared, % Black, % Hispanic, % White, % with less than high school degree, and the number of dentists per capita were all averaged for the pre-policy period (2006-2008). [a] Control states for the low-income sample included 43 states: Alabama, Arkansas, California, Colorado, Connecticut, Delaware, District of Columbia, Florida, Georgia, Hawaii, Idaho, Illinois, Indiana, Iowa, Kansas, Kentucky, Louisiana, Maryland, Massachusetts, Mississippi, Missouri, Montana, Nebraska, Nevada, New Hampshire, New Jersey, New Mexico, New York, North Carolina, North Dakota, Ohio, Oklahoma, Pennsylvania, Rhode Island, South Carolina, South Dakota, Tennessee, Texas, Utah, Virginia, West Virginia, Wisconsin, Wyoming.

This supplemental material has been provided by the authors to give readers additional information about their work.

eTable 6. Predictor Balance in the Prepolicy Period in the Medicaid Sample (2006-2008)

	Minnesota	Synthetic Minnesota	Average of control units[a]
**Variable**			
**All adults**			
Age	36.4	36.9	36.8
Age squared	1501.3	1527.1	1520.1
% Black (log)	-2.1	-2.6	-2.6
% Hispanic (log)	-3.2	-1.5	-2.0
% White (log)	-0.29	-0.70	-0.69
% with less than high school degree	3.1	8.8	7.3
Number of dentists per capita	60.1	65.2	58.6
Prevalence of dental visit in previous year, 2006	57.3	57.3	51.6
Prevalence of dental visit in previous year, 2008	59.3	59.3	52.0
**White subpopulation**			
Age	37.4	36.9	37.4
Age squared	1579.5	1540.5	1579.4
% with less than high school degree	2.9	2.9	2.9
Number of dentists per capita	60.1	62.6	58.6
Prevalence of dental visit in previous year, 2006	57.0	57.1	51.9
Prevalence of dental visit in previous year, 2008	56.3	56.3	52.3
**Non-White subpopulation**			
Age	33.6	35.5	35.5
Age squared	1265.5	1395.3	1408.0
% with less than high school degree	3.4	12.8	11.4
Number of dentists per capita	60.1	71.5	58.6
Prevalence of dental visit in previous year, 2006	58.1	57.6	51.6
Prevalence of dental visit in previous year, 2008	69.5	64.3	52.3

Note. Age, age squared, % Black, % Hispanic, % White, % with less than high school degree, and the number of dentists per capita were all averaged for the pre-policy period (2006-2008). [a] Control states for the Medicaid sample included 19 states: Arkansas, Connecticut, District of Columbia, Indiana, Iowa, Kentucky, Massachusetts, Nebraska, New Jersey, New Mexico, New York, North Carolina, North Dakota, Ohio, Pennsylvania, Rhode Island, South Dakota, Wisconsin, Wyoming.

This supplemental material has been provided by the authors to give readers additional information about their work.

© 2022 Elani et al. *JAMA Health Forum.*

eFigure 1. Placebo Analysis for the Full Sample for All Control States Before and After the Adoption of Dental Therapy in Minnesota in 2009

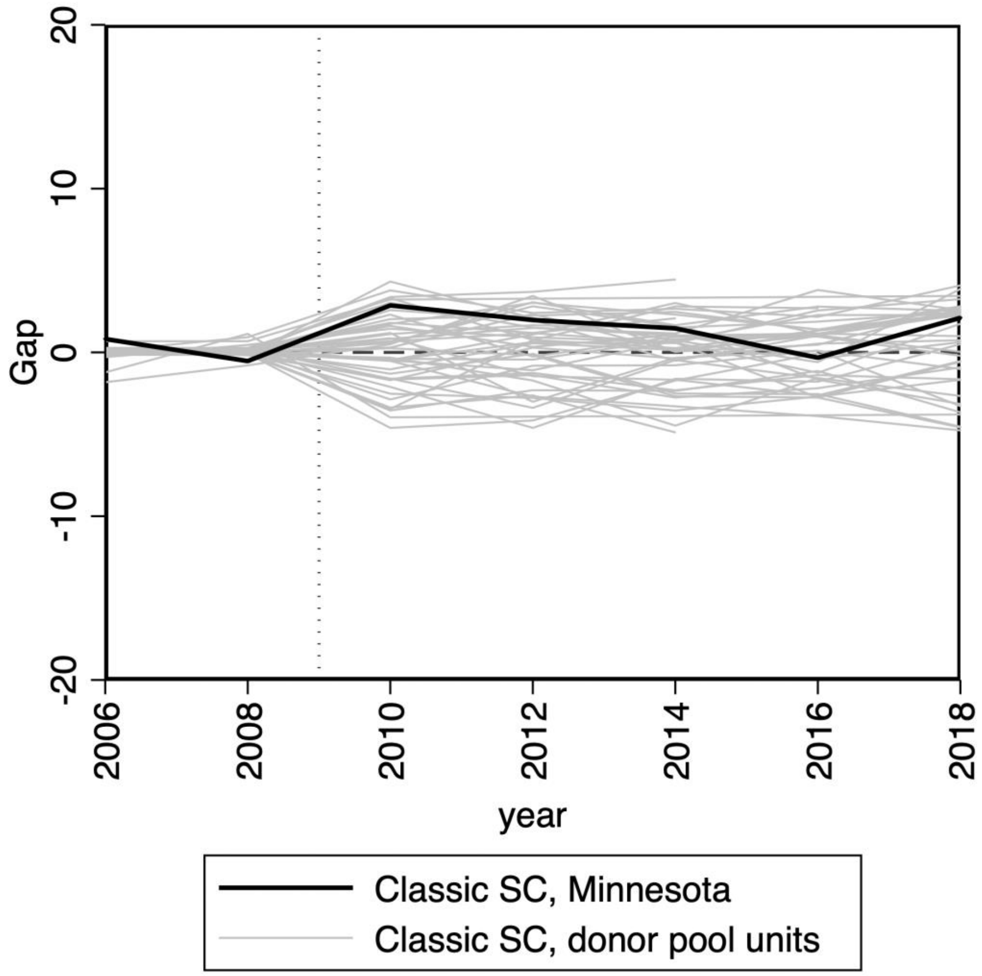

This supplemental material has been provided by the authors to give readers additional information about their work.

© 2022 Elani et al. *JAMA Health Forum.*

eFigure 2. Placebo Analysis for the Low-Income Sample for All Control States Before and After the Adoption of Dental Therapy in Minnesota in 2009

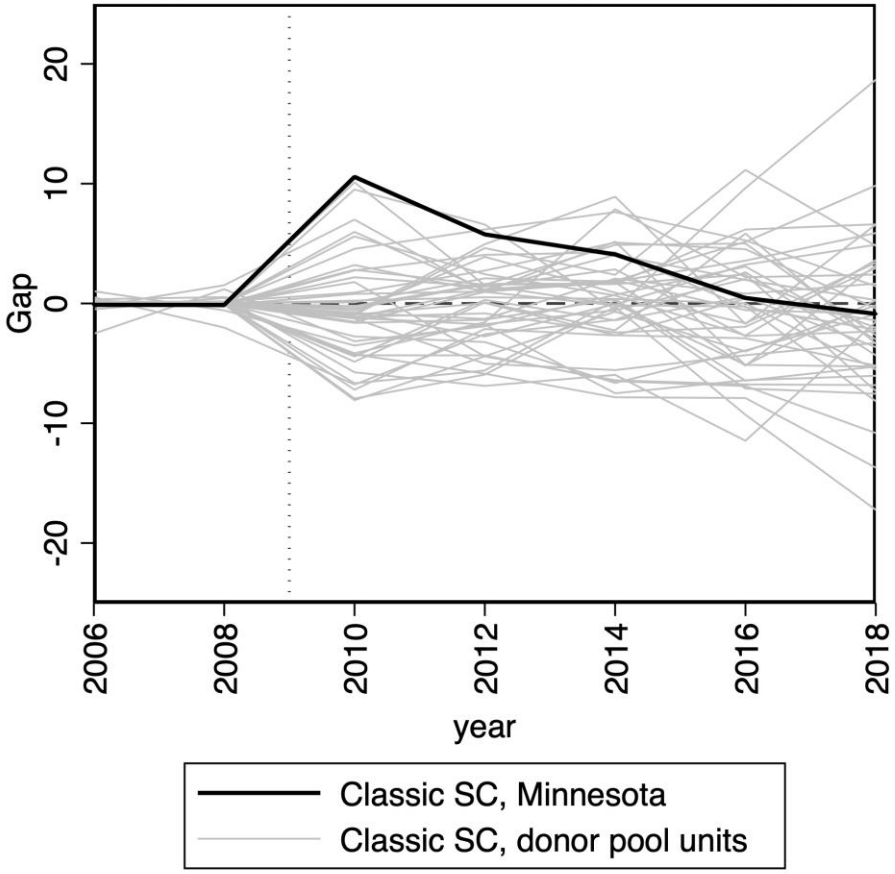

This supplemental material has been provided by the authors to give readers additional information about their work.

© 2022 Elani et al. *JAMA Health Forum.*

eFigure 3. Placebo Analysis for the Medicaid Sample for All Control States Before and After the Adoption of Dental Therapy in Minnesota in 2009

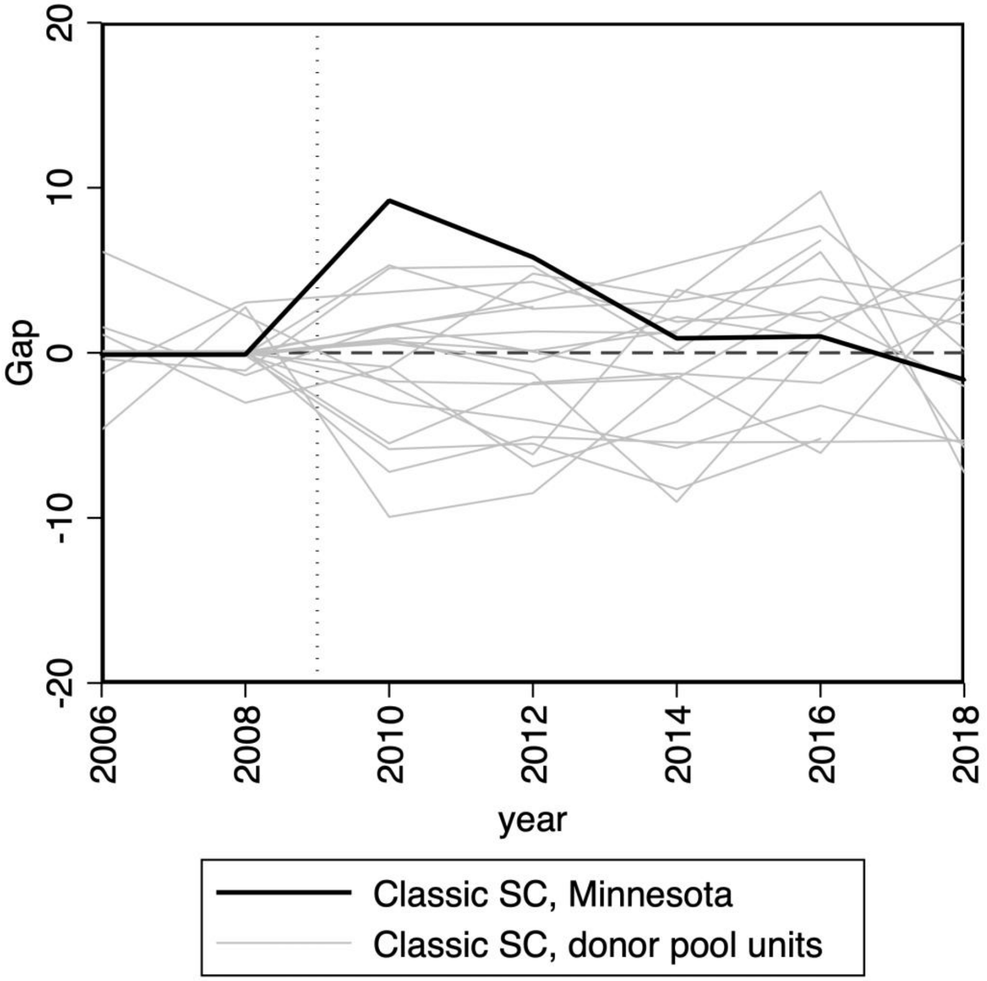

This supplemental material has been provided by the authors to give readers additional information about their work.

© 2022 Elani et al. *JAMA Health Forum.*
